# Gender-informed psycho-educational programme to promote respectful relationships and reduce postpartum common mental disorders among primiparous women: long-term follow-up of participants in a community-based cluster randomised controlled trial

**DOI:** 10.1017/gmh.2018.20

**Published:** 2018-09-25

**Authors:** Jane Fisher, Thach Tran, Karen Wynter, Harriet Hiscock, Jordana Bayer, Heather Rowe

**Affiliations:** 1 Jean Hailes Research Unit, School of Public Health and Preventive Medicine, Monash University, Victoria, Australia; 2 Murdoch Childrens Research Institute, Victoria, Australia; 3 Centre for Community Child Health, The Royal Children's Hospital, Victoria, Australia; 4 Department of Paediatrics, University of Melbourne, Victoria, Australia; 5 School of Psychology and Public Health, La Trobe University, Victoria, Australia

**Keywords:** Interventions, postnatal common mental disorders, prevention, psychoeducation

## Abstract

**Background:**

What Were We Thinking (WWWT) is a gender-informed, psychoeducational programme to promote respectful relationships and skilled management of unsettled infant behaviours and thereby reduce postpartum common mental disorders. It comprises a highly structured seminar for couples and babies, usual primary care from a WWWT-trained nurse and take-home print materials. The aim was to assess long-term outcomes after a cluster randomised controlled trial of WWWT.

**Method:**

Trial participants who consented completed a computer-assisted telephone interview 18 months postpartum. Depressive symptoms were assessed with the Patient Health Questionnaire (PHQ-9) and anxiety symptoms with the Generalised Anxiety Disorder Scale (GAD-7). Impacts of baseline characteristics and trial arm on changes in scores from baseline to follow-up were calculated using Conditional Latent Growth Curve Models adjusting for prognostic indicators and controlling for clustering effects.

**Results:**

Overall, 314/400 (78.5%) women contributed data at baseline (6 weeks postpartum), trial endline (26 weeks postpartum) and follow-up (12 months after trial endline). In intention-to-treat analyses, there was a significantly greater improvement in adjusted GAD-7 scores [regression coefficient (RC) −0.55; 95% confidence interval (CI) −0.94 to −0.17] and non-significant improvement (RC −0.27; 95% CI −0.63 to 0.08) in PHQ-9 scores from baseline to follow-up in the intervention than the control arm. In a per-protocol analysis, the proportion with GAD-7 scores ⩽4 (asymptomatic) improved 24.1% (55.7% baseline to 79.8% follow-up, *p* = 0.043) among women who received the full WWWT programme, which included the seminar, compared with 2.4% (77.1–79.5%, *p* = 0.706) among those who received the partial intervention (usual care from WWWT-trained nurse and print materials).

**Conclusions:**

The WWWT programme has a significant sustained beneficial impact on postnatal generalised anxiety among primiparous women compared with usual care. The in-person seminar is the most influential component of the intervention. Psycho-educational programmes integrated into primary care appear promising as a strategy to reduce postpartum common mental disorders.

## Introduction

The Global Strategy for Women's, Children's and Adolescents’ Health (2016–2030) identifies explicitly that every woman has the right to mental health and well-being (WHO, 2015). Mental health problems among women who are pregnant or have recently given birth are a global public health priority (WHO, 2018). They are associated with reduced quality of life, and participation in health care, and increased risks of adverse birth outcomes among women and, in particular in resource-constrained contexts, with compromised early development among their children (Fisher *et al*. 2010*a*, 2011; Black *et al*. 2017). There is a call for cost-effective programmes that can be integrated into primary maternal and child health care to reduce these problems (Rahman *et al*. 2013).

Traditional approaches to understanding perinatal mental health problems have conceptualised them as being intrinsic to women, and as having adverse consequences for those around them, in particular their intimate partners and their babies. The notion that a woman's psychological state might reflect day-to-day interactions within these relationships and that they might be a promising target for intervention is less common (Fisher *et al*. 2010*b*; Rowe & Fisher, 2010*b*; Fisher *et al*. 2016).

### Potentially modifiable risks for postnatal mental health problems

#### Relationship with the intimate partner

While the relationship with an intimate partner is well established as a central determinant of a woman's perinatal mental health, the qualities that might be influential have not been characterised precisely. The Scottish Intercollegiate Guidelines Network completed a systematic review to inform the clinical guideline for the management of perinatal mood disorders and concluded that ‘poor partner relationship’ has moderate-to-strong associations with postnatal depression (Scottish Intercollegiate Guidelines Network, 2012).

In 2013, Howard *et al*. (2013) contributed the first systematic review of the evidence about intimate partner violence and perinatal mental health problems. In a meta-analysis of the few available cohort studies, there was a threefold increase in the likelihood of clinically significant symptoms of postnatal depression [odds ratio (OR) 3.1, 95% confidence interval (CI) 2.7–3.6] among women who had experienced intimate partner violence during pregnancy compared with those who had not. There was consistent evidence that having ever experienced intimate partner violence increased the likelihood of having postnatal depression, anxiety and post-traumatic stress disorder symptoms. However, in this review while the period of exposure (lifetime, prior year or during pregnancy) was examined, the nature of the violence perpetrated by an intimate partner was not reported.

We demonstrated among a consecutive cohort of women admitted to a residential early parenting programme in Australia that those who had clinically significant depressive symptoms were more likely than those without to experience their partners as coercive, critical and controlling and to have babies who they felt unable to settle (Fisher *et al*. 2002). Similarly, in a community-based cohort study of 161 Australian, primiparous women and their partners from diverse geographical and socio-economic circumstances, those who had experienced their partners as critical, coercive or intimidating 6 weeks postpartum, had significantly more depressive symptoms 6 months postpartum than others. Symptoms were more severe among women with the vulnerable personality traits of having high interpersonal sensitivity and limited assertiveness, and whose infants cried and fussed for prolonged periods (Wynter *et al*. 2014).

Together these suggest that there might be a spectrum of problematic qualities in the intimate partner relationship that increase the risk of postnatal mental health problems, ranging from limited practical and emotional support to emotionally abusive coercive behaviours and physical and sexual abuse.

#### Unsettled infant behaviours

The birth of a sick or premature baby increases the risk of postnatal mental health problems (Hagan *et al*. 2004). The impact on perinatal mental health of the temperament or behaviour of healthy babies is however less well understood, but there are indicators. Mothers of infants who cry excessively report significantly more parenting stress and depressive symptoms (Radesky *et al*. 2013), and less sense of competence and efficacy compared with other mothers. They are less likely to experience their infants as a source of positive reinforcement. Excessive, inconsolable infant crying and resistance to comforting have been associated with earlier cessation of breastfeeding, frequent changes of infant formula, maternal irritability, poorer mother–infant relationship and heightened risk of infant abuse (Wolke *et al*. 1994; Lehtonen *et al*. 2000). If the infant wakes frequently and sleeps only for short periods, risk of severe fatigue among caregivers is increased. Mothers of infants who sleep poorly have worse self-reported physical and mental health than mothers of infants who sleep well (Bayer *et al*. 2007).

#### Occupational fatigue

Occupational fatigue is a state of work-related mental or physical exhaustion accompanied by poor judgement, slower reactions to events, increased clumsiness, reduced concentration and vigilance and impaired memory, which have an adverse effect on safety sensitivity (Van der Linden *et al*. 2003). Severe occupational fatigue leads to increased irritability, agitation, reduced empathy and sociability, low mood, and a general loss of insight and self-awareness (Rogers & Grunstein, 2005).

### Universal interventions to prevent postnatal mental health problems

There is a greater public benefit from preventing health problems from occurring or recurring than providing curative care. Universal interventions that include all eligible citizens are preferred, because they are less stigmatizing, more likely to be used and even small reductions in population prevalence have a greater public health benefit than treating people who already have symptoms (Mrazek & Haggerty, 1994). Conceptual models of the links between risk and protective factors and health outcomes underpin all prevention efforts. The conceptualisation of potentially modifiable risk and protective factors for postnatal mental health is reflected in the approaches taken to prevention.

Seven universal interventions to prevent postnatal depression tested in randomised controlled trials were published prior to 2016. None specified the risk factor being addressed, or whether it was being targeted directly or indirectly (Fisher *et al*. 2016). Their impact was limited; only intensive midwife home-visiting (MacArthur *et al*. 2002; MacArthur *et al*. 2003) was associated with reduced symptoms (Edinburgh Postnatal Depression Scale mean scores and proportion scoring in the clinical range assessed 4 and 12 months postpartum) in the intervention compared with the control group. This could have been because the only outcomes assessed were depressive symptoms and not anxiety and adjustment disorders, which might have been reduced, but were not detected. Analyses did not take potential prognostic indicators into account. None of the interventions were gender-informed, included the intimate partner or the baby(ies) or addressed occupational fatigue (Fisher *et al*. 2016).

### The What Were We Thinking programme

The What Were We Thinking (WWWT) programme represents a new way of thinking about prevention of postnatal common mental disorders (PCMD). It is based on Brown and Harris's social theory (Brown & Harris, 1978) that depression arises in contexts of humiliation and entrapment (Fisher *et al*. 2010*b*; Rowe & Fisher, 2010*a*); Beck's theory that anxiety develops as a result of helplessness and lack of agency (Beck *et al*. 1985) and health promotion theories that potentially modifiable risks for these experiences can be addressed directly (Rowe & Fisher, 2010*b*). WWWT is predicated on principles that interactions between intimate partners can be modified to become more respectful by decreasing critical, controlling behaviours and increasing collaboration, shared problem-solving and mutual appreciation; and that interactions with babies can be improved by strengthening capabilities to manage unsettled infant behaviours. The programme takes a psycho-educational approach providing evidence-based, life-stage-specific active learning opportunities, with accessible information for ongoing reference. These give participants the understanding, language and skills to adapt to changed roles and responsibilities, resolve conflict respectfully, provide competent effective infant care and reduce fatigue. Language used in all components of the WWWT intervention is specifically chosen to challenge gender stereotypes about roles and responsibilities, position mothering and fathering as of equal importance, respect the unpaid workload and improve emotional literacy without the use of psychiatric labelling. The programme comprises a face-to-face small group highly structured seminar for couples and their babies, take-home materials for ongoing reference and usual primary care from a WWWT-trained maternal and child health nurse (MCHN) [see Box 1 reproduced from Fisher *et al*. (2016) for a summary of theory, content and structure].

Box 1. The What Were We Thinking (WWWT) programme

WWWT is a highly structured, gender-informed, interactive psychoeducational programme for couples and their first baby.

*Theoretical principles*

- Improvements in day-to-day interpersonal interactions within families are of fundamental importance to preventing common mental disorders;
- Partner and infant behaviours can be modified to decrease those that contribute to psychological distress and increase those that promote confidence and sense of competence;
- Women prefer to receive emotional care and practical support within their intimate relationships than increased care from health professionals;
- Depressive and anxiety disorders are not easily distinguished and prevention strategies should use a trans-diagnostic approach;
- Readily understood, evidence-informed knowledge and opportunities for active learning and skills development need to be made available at the critical developmental stage at which they are needed;
- A psycho-educational approach addresses plausible psychological mechanisms using education to meet salient learning needs;
- Language used in the intervention is crucial and needs to challenge gender stereotypes, position mothering and fathering as different but of equal importance, respect the unpaid workload and name and normalise emotions without the use of psychiatric labelling;
- Women's experiences of humiliation can be reduced by increasing their partners’ appreciation and empathy, and reducing critical and controlling behaviours;
- Experiences of entrapment can be countered by promoting infant care as a shared endeavour in which parents with comparable competence can permit each other independent or shared leisure;
- Cognitive, rather than emotion-focused, responses to infant crying can be promoted by building skills to respond actively and effectively, rather than avoidantly;
- Occupational fatigue among parents is minimised by teaching them how to understand and promote adequate infant sleep using evidence-informed behaviour management strategies;
- Together these lead to increased confidence and competence, and reduced depression, anxiety and adjustment disorders.

Content and structure

WWWT has an educational framework, comprising structured, easily comprehended learning activities made available at a critical life stage when parenting-specific learning needs are high. It has three interlinked components:

- Primary care from a WWWT-trained maternal and child health nurse
  Primary care is provided by maternal and child health nurses who have been trained in programme theory and implementation.
- Print materials
  Attractively illustrated programme materials in accessible plain language including worksheets for each learning activity and a short book.
- Face-to-face seminar offered at 6–8 weeks postpartum

Small group sessions for about five couples and their babies are integrated into a standard primary care programme and offered in a short single-day programme on a Saturday to maximise access. The sessions have two sections:

- *About Babies* includes learning activities about infant temperament, crying and fussing, recognition of tired cues, sleep needs, establishing feed–play–sleep routines of daily care and safe, sustainable settling strategies: collectively known as ‘infant behaviour management’.
- *About Parents* includes learning activities about differences between how parenthood had been imagined and is being experienced; recalling the difficult and pleasing aspects of the baby**’** s birth; recognizing, naming and renegotiating the unpaid workload fairly in non-confrontational ways; acknowledging the disenfranchised losses of parenthood as well as the gains; identifying experiences within parents’ families of origin that they wish to duplicate or to relinquish; and identifying gaps in support.

Adult learning strategies, including group discussion, focused tasks to be undertaken individually using the print materials and then discussed as a couple; practice in problem-solving and negotiation; hands on supported practice in infant wrapping and settling; short talks and practical demonstrations. (Reproduced from Fisher *et al*. 2016).

In Victoria, Australia where this trial was conducted, all first-time mothers are entitled to attend first-time parents’ groups at their local maternal and child health centre (MCHC). We wanted to establish whether the WWWT programme could be incorporated into that established primary care programme. We completed a cluster randomised controlled trial (cRCT) of WWWT in six local government areas in Victoria, Australia (Fisher *et al*. 2016). MCHCs, the clustering unit, were allocated randomly to provide the usual standard of care or this care plus the WWWT programme. The WWWT seminar, facilitated by MCHNs, was integrated into the routine first-time parents’ groups programme and run on a Saturday to maximise men's participation. Mean fidelity scores for the About Babies section were: delivery quality 4.6/5 and participant engagement 4.4/5 and About Parents were: 3.8/5 and 3.4/5. The primary outcome was any depressive, anxiety or adjustment disorder in the prior 30 days ascertained by Composite International Diagnostic Interviews and scores on the Generalised Anxiety Disorder seven-item scale (GAD-7) and the Patient Health Questionnaire nine-item scale (PHQ-9) scores (Spitzer *et al*. 1999; Spitzer *et al*. 2006).

All primiparous women allocated to receive routine care at the MCHCs were eligible to participate in the study. Overall, 400 primiparous women <6 weeks postpartum, sufficiently literate and fluent in English to comprehend participant information, give consent and complete telephone interviews, were recruited. All data were collected in individual computer-assisted telephone interviews at baseline (6 weeks) and endline (26 weeks) postpartum. Participation by men in routine postpartum primary care remains rare in Australia and fewer than half (46%) of the eligible and invited couples actually attended the seminar together, most commonly because men were not willing to come. All participants in the intervention arm were given print materials either at the seminar or by post. In intention-to-treat (ITT) analyses controlling for prognostic indicators, the adjusted OR (AOR) of PCMD in the intervention compared with the usual care group was 0.78 (95% CI 0.38–1.63, ns). However, mild–to-moderate anxiety symptoms (AOR 0.58, 95% CI 0.35–0.97) and poor self-rated health, an indicator of fatigue (AOR 0.46, 95% CI 0.22–0.97), were significantly lower. In a per-protocol (PP) analysis comparing outcomes among those who received the full (three-component) intervention and usual care groups, the AOR of PCMD was 0.36 (95% CI 0.14–0.95). Receiving only the partial intervention was not associated with a reduction in the primary outcome indicating that the seminar was the influential element of the programme (Fisher *et al*. 2016).

We undertook ancillary exploratory analyses with the same statistical models used for comparisons between trial arms, to elucidate the findings. Participants were categorised into three groups: (i) those who met criteria for PCMD, (ii) those who had scores ⩾5 (cut-off for clinically significant symptoms) on either PHQ-9 or GAD-7, but did not meet onset, disability or core symptom criteria for adjustment disorders, and (iii) women with no clinically significant symptoms. There was a significantly lower prevalence of mild-to-moderate symptoms of depression and anxiety in the intervention than the control group. Babies unsettled at baseline had significantly fewer unsettled behaviours 6 months postpartum in the intervention than the usual care group and their parents were more likely to be adhering to recommendations about safe sleeping place. Among those whose intimate partner relationship was more optimal [Intimate Bonds Measure (IBM; Wilhelm & Parker, 1988) Care Subscale scores ⩾75th percentile and Control Subscale scores ⩽25th percentile of sample distribution] at baseline, participation in WWWT was associated with significantly fewer emotionally abusive behaviours at endline (Fisher *et al*. 2016).

In anonymously completed post-seminar surveys, the WWWT seminar was appraised as salient, comprehensible and useful by more than 85% of participants. No harms to breastfeeding or mother–infant relationship were detected. Including all the costs of developing the programme, it was found to be cost-effective. A smaller number of women were admitted to residential early parenting programmes for treatment of maternal mental health problems or unsettled infant behaviours in the intervention than the control arm (Ride *et al*. 2016).

The aims were to (i) establish whether there was a long-term impact on primiparous women's mental health of the WWWT programme and (ii) identify baseline characteristics associated with this outcome.

## Methods

### Study design

A follow-up of participants in the cRCT of the WWWT intervention (Fisher *et al*. 2016).

### Data sources

All data were collected in individual structured interviews, which included standardised instruments and study-specific fixed-response items.

**Women's mental health** was assessed at all waves using the PHQ-9 to assess symptoms of major depression, and the GAD-7 to assess anxiety symptoms. Both use symptom criteria from the Diagnostic and Statistical Manual of Mental Disorders, Fourth Edition (DSM-IV) (Spitzer *et al*. 1999; Spitzer *et al*. 2006). Each item on these scales has four response options to a question asking whether in the past 2 weeks the symptom has been experienced: ‘not at all’, ‘on several days’, ‘more than half the days’, or ‘nearly every day’, scored 0–3. Total scores on the PHQ-9 range from 0 to 27 and on the GAD-7 from 0 to 21. On both scales, scores from 0 to 4 indicate that there are no symptoms, from 5 to 9 mild symptoms, from 10 to 14 moderate symptoms, from 15 to 19 moderately severe symptoms and of 20 and above that symptoms are severe.

**The quality of relationship between intimate partners** was assessed at baseline using the IBM, which is regarded as a stable indicator. It comprises two subscales: **Care** (12 items) assesses sensitivity, warmth, emotional responsiveness, trust, physical gentleness and kindness and **Control** (12 items) assesses coercion, dominance, exertion of power and extent of criticism (Wilhelm & Parker, 1988). IBM Care has a Cronbach's *α* = 0.94 and correlation with clinical interview ratings of quality of relationship = 0.68; IBM Control has a Cronbach's *α* = 0.89 and correlation with clinical interview ratings of quality of relationship = 0.74 (Wilhelm & Parker, 1988).

**Socio-demographic characteristics** including age, marital status, highest level of completed education, occupation, country of birth, language spoken at home and whether or not a health-care card (an indicator of being in a low socio-economic position and receiving social protection benefits) is held were assessed using study-specific fixed-choice questions at baseline.

**Indicators for other risk and protective factors for postnatal mental health problems** collected at baseline included:

- *Childhood maltreatment*: experience of any physical and/or sexual abuse before the age of 16 years assessed using two study-specific fixed-choice questions: (1) Before the age of 16, were you ever hit, punched, beaten or otherwise physically mistreated by a member of your family, a step-parent or a person in authority? and (2) Before the age of 16, did you experience sexual contact from a family member or person in authority?
- *Personality*: scores on the Vulnerable Personality Style Questionnaire (VPSQ) Vulnerability Subscale (six items) which measures oversensitivity to the opinions of others, timidity and lack of assertiveness (Boyce *et al*. 2001).
- *Unsettled infant behaviours*: assessed using questions derived from the Barr Parental Diary (Barr *et al*. 1988) about how many hours the infant fussed/cried in the prior 24 h, from which we derived a binary variable: crying/fussing ⩾ or <3/24 h, and a single question which is an established indicator of day and night time sleep problems: ‘Over the last 2 weeks, has your baby's sleep generally been a problem for you?’ (Hiscock *et al*. 2007).
- *Psychiatric history*: lifetime history of having been diagnosed with a common or severe mental disorder.

### Procedure

Twelve months after the trial endline interview, when infants were about 18 months old, all women who had given consent to be informed about a follow-up study were contacted by their preferred method (email, SMS, telephone call or letter) and invited to participate. All those who consented completed an individual computer-assisted telephone interview with a trained, supervised health research assistant blinded to group allocation, at a time that was convenient to the participant. Protocols were available for disclosure of suicidal ideas or severe symptoms.

### Data management and statistical analyses

All data were entered in a password-protected project-specific database. We followed Gupta's (2011) recommendation that because non-compliance is common in randomised controlled trials, both ITT and PP analyses should be used to examine the impact of an intervention in a superiority trial. Gupta argues that in ITT analyses, estimates of intervention effects are generally conservative because of dilution due to non-compliance and that PP analyses are more accurate indicators of impact. We minimised bias by following up all participants whether or not they had received the intervention.

Analyses were conducted in two stages.

In the first stage, descriptive analyses and univariable comparisons were completed. Continuous variables were described using mean and standard deviation values for those that were normally, or median and interquartile range values for those that were non-normally distributed. The distributions of PHQ-9 and GAD-7 and the differences in proportions of clinically significant symptoms between baseline and follow-up (the magnitude in changes) were compared between intervention and control arms. Similar comparisons were made between women who received the partial (primary care from a WWWT-trained MCHN and print materials) and the full three-component intervention, which included attending the seminar. Statistical procedures: *t* tests (for normally distributed continuous), the Wilcoxon rank-sum tests (for non-normally distributed continuous) and χ^2^ tests (for categorical variables) were used to make these comparisons as appropriate. Descriptive analyses were performed in Stata version 14 (StataCorp, 2015).

In the second stage, the impacts of baseline characteristics and trial arm on the changes of PHQ-9 and GAD-7 scores from 6 weeks to 18 months postpartum were assessed using separate Conditional Latent Growth Curve Models controlling for prognostic indicators. The outcomes, GAD-7 and PHQ-9 scores, were modelled as growth curves with two latent variables including the intercept (the mean score at baseline) and slope (the mean change in scores from 6 weeks to 18 months). The associations between covariates and the intercept and slope were identified simultaneously. The coefficients of the associations are interpreted as linear regression coefficients (RCs). As the distributions of the GAD-7 and PHQ-9 scores were skewed, the models were estimated using maximum likelihood parameter estimates with standard errors, which are robust to non-normality. Growth curve analysis was performed in Mplus version 7.4 (Muthén & Muthén, 2014) using maximum likelihood estimates for clustered data. The standard errors of estimations and χ^2^ test of model fit were computed taking clustering into account. In order to evaluate model fit, we used root mean square error of approximation (RMSEA) with values <0.05 indicating a good fit, and Tucker–Lewis index and comparative fit index with values >0.95 indicating a good fit (Hu & Bentler, 1999).

The distributions of the GAD-7 and PHQ-9 scores are skewed, so we performed sensitivity analyses with transformations of these scores. We used the ‘ladder’ command in Stata to search for the best transformation to convert these scores into approximately normally distributed variables. The transformed outcomes were modelled as growth curves and the associations between covariates and the intercept and slope were identified simultaneously. The coefficients are in a transform unit, and are therefore difficult to interpret. These analyses were used to confirm the significances of covariates and the GAD-7 and PHQ-9 trajectories that had been found in the growth modelling analyses of the raw data.

Only data from women who contributed data at baseline (6 weeks), trial endline (26 weeks) and follow-up (18 months postpartum) were included in analyses in the first stage. In the second stage, data from all 400 participants were included and missing data were handled by the full information maximum likelihood (Enders & Bandalos, 2001; Muthén & Muthén, 2014; StataCorp, 2015).

## Results

### Participants

**Fig. 1.**
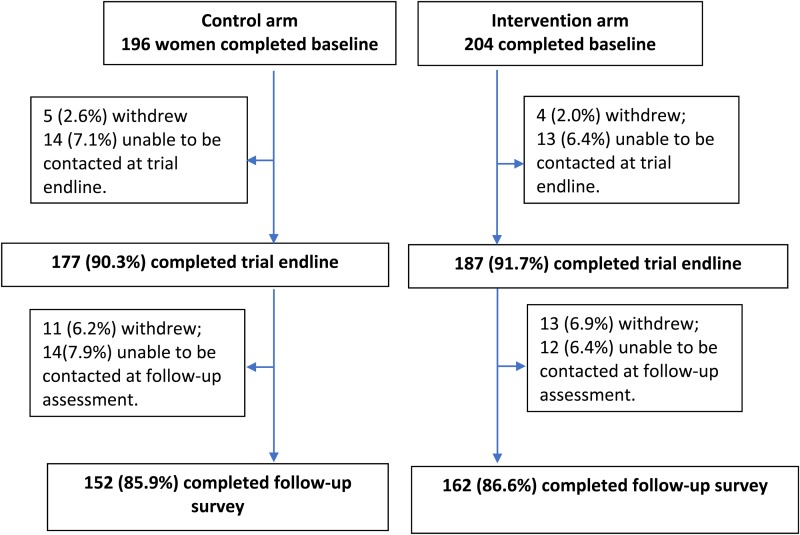
Study profile.

Overall, 400 women contributed data to this study (Fig. 1). Among them, 314 (78.5%) provided complete data and 86 who had provided baseline data were not contactable or withdrew at trial endline or follow-up assessment waves. The baseline characteristics of women who provided complete and incomplete data are presented in Table 1. There were some differences between the two groups in terms of socio-demographic characteristics: more women who provided complete data had completed post-secondary education and fewer held a health-care card than women who provided only baseline data. However, there were no differences in baseline mental health indicators. Women were on average 18.8 (s.d. 1.2) months postpartum at follow-up.

**Table 1.** Baseline characteristics of women who provided complete and incomplete data

	Complete (*N* = 314)	Incomplete^a^ (*N* = 86)	*p* value
Socio-demographic			
Age (years) , mean <s.d.>	32.1<5.0>	31.1<5.4>	0.124
Born in Australia, *n* (%)	246 (78.3)	60 (69.7)	0.097
Speak only English at home, *n* (%)	266 (84.7)	67 (77.9)	0.134
Holds a health-care card^b^, *n* (%)	36 (11.5)	19 (22.1)	0.011
Married, *n* (%)	228 (72.6)	55 (64.0)	0.083
Highest education level, *n* (%)			0.002
University degree or above	206 (65.6)	43 (50.0)	
Post-secondary trade training or certificate	61 (19.4)	16 (18.6)	
Up to or complete secondary schooling	47 (15.0)	27 (31.4)	
Managerial or professional occupation	175 (55.7)	32 (37.2)	0.002
*Other risk and protective factors for postnatal mental health*			
Childhood maltreatment, *n* (%)			
Physical abuse	21 (6.6)	8 (9.3)	0.407
Sexual abuse	6 (1.9)	2 (2.3)	0.811
Vulnerable Personality Scale			
Vulnerability Subscale score, median [IQR]	12 [9–15]	11 [9–15]	0.489
Intimate Bonds Measure			
Care Subscale score, median [IQR]	34 [32–36]	35 [33–36]	0.779
Control Subscale score, median [IQR]	4 [2–6]	4 [2–6]	0.769
Mental health			
PHQ-9 score, median [IQR]	3 [2–5]	2.5 [1–5]	0.934
GAD-7 score, median [IQR]	2 [1–5]	2 [0–5]	0.677
Psychiatric history, *n* (%)	79 (25.1)	19 (22.1)	0.558

a Withdrew at trial endline or at follow-up.

b Health-care cards are held by people whose main income is a social protection benefit, who are unwaged students or have a very low household income.

IQR, interquartile range.

### Trajectories of depressive and anxiety symptoms from 6 weeks to 18 months postpartum

The GAD-7 and PHQ-9 scores among the women who contributed complete data diminished over the three time points, but these changes were not statistically significant (Tables 2 and 3). In the Conditional Latent Growth Curve Models, the slopes of the GAD-7 and PHQ-9 growth trajectories are negative when socio-demographic and risk factors at baseline are taken into account.

**Table 2.** Conditional Latent Growth Curve Model of women's GAD-7 scores from 6 weeks to 18 months postpartum

	Coefficient^a^	95% CI
GAD-7 trajectories		
Mean GAD-7 score at 6 weeks postpartum^b^	**3**.**33**	**[0.98–5.68]**
Change in GAD-7 score from 6 weeks to 18 months postpartum^c^	−0.24	[−1.63 to 1.15]
Characteristics associated with GAD-7 score at baseline (linear regression)		
Trial arm (intervention cf. control)	**0**.**88**	**[0.27–1.48]**
Age (in years)	0.01	[−0.07 to 0.07]
Language spoken at home (only English cf. other languages)	−0.82	[−1.69 to 0.04]
Education level (university degree cf. partial or complete secondary or certificate level)	0.16	[−0.54 to 0.86]
Marital status (married cf. *de facto* or single)	0.33	[−0.43 to 1.09]
Occupation (managerial or professional cf. other occupations)	0.12	[−0.52 to 0.75]
Holds a health-care card (yes cf. no)	0.41	[−0.38 to 1.20]
VPS Vulnerability Subscale score	**0**.**37**	**[0.30–0.44]**
Past psychiatric illness (yes cf. no)	0.62	[−0.06 to 1.30]
Childhood physical/sexual abuse (yes cf. no)	0.69	[−0.61 to 1.99]
Number of pregnancies (one cf. >1)	−0.08	[−0.60 to 0.44]
Duration of baby's crying/fussing prior 24 h (≥3 cf. <3 h)	−0.09	[−0.79 to 0.62]
Baby's sleep a problem in past 2 weeks (yes cf. no)	**1**.**98**	**[1.07–2.88]**
IBM Care score	0.11	[−0.70 to 0.92]
IBM Control score	0.63	[−0.16 to 1.42]
Characteristics predicting change of GAD-7 scores from baseline to follow-up (linear regression)		
Trial arm (intervention cf. control)	−**0**.**55**	[−**0.94 to −0.17]**
Age (in years)	0.01	[−0.05 to 0.05]
Language at home (English cf. others)	0.37	[−0.03 to 0.78]
Education level (university degree cf. partial or complete secondary or certificate level)	−0.08	[−0.59 to 0.43]
Marital status (married cf. *de facto* or single)	0.04	[−0.32 to 0.40]
Occupation (managerial or professional cf. other occupations)	0.07	[−0.30 to 0.43]
Holds a health-care card (yes cf. no)	0.37	[−0.19 to 0.92]
VPS Vulnerability Subscale score	−**0**.**10**	[−**0.15 to −0.06]**
Past psychiatric illness (yes cf. no)	0.28	[−0.15 to 0.70]
Childhood physical or sexual abuse (yes cf. no)	−0.19	[−0.84 to 0.47]
Number of pregnancies (one cf. >1)	0.17	[−0. 18 to 0.51]
Duration of baby's crying/fussing prior 24 h (≥3 cf. <3 h)	0.06	[−0.33 to 0.43]
Baby's sleep a problem in past 2 weeks (yes cf. no)	−**0**.**56**	[−**1.04 to −0.08]**
IBM Care score	−0.12	[−0.54 to 0.30]
IBM Control score	−0.34	[−0.98 to 0.31]
**Fit indices**	**Estimates**	
RMSEA (probability RMSEA ⩽0.05)	0.025 (0.90)	
Comparative fit index	0.987	
Tucker–Lewis index	0.960	

a All regression coefficients were estimated simultaneously using Conditional Latent Growth Curve Model. Significant regression coefficients are in bold.

b Intercept.

c Slope.

**Table 3.** Conditional Latent Growth Curve Model of women's PHQ-9 scores from 6 weeks to 18 months postpartum

	Coefficient^a^	95% CI
PHQ-9 trajectories		
Mean PHQ-9 score at 6 weeks postpartum^b^	**3**.**67**	**[1.19 to 6.19]**
Change in PHQ-9 score from 6 weeks to 18 months postpartum^c^	−0.12	[−1.61 to 1.37]
Characteristics associated with PHQ-9 scores at baseline (linear regression)		
Trial arm (intervention cf. control)	0.50	[−0.04 to 1.05]
Age (in years)	0.01	[−0.06 to 0.08]
Language spoken at home (only English cf. other languages)	−**1**.**55**	[−**2.29 to −0.80]**
Education level (university degree cf. partial or complete secondary or certificate level)	−0.14	[−0.70 to 0.43]
Marital status (married cf. *de facto* or single)	0.64	[−0.05 to 1.34]
Occupation (managerial or professional cf. other occupations)	−0.05	[−0.63 to 0.52]
Holds a health-care card (yes cf. no)	0.89	[−0.04 to 1.83]
VPS Vulnerability Subscale score	**0**.**22**	**[0.15–0.30]**
Past psychiatric illness (yes cf. no)	**0**.**80**	**[0.09–1.51]**
Childhood physical/sexual abuse (yes cf. no)	0.90	[−0.32 to 2.11]
Number of pregnancies (one cf. >1)	−0.18	[−0.70 to 0.33]
Duration of baby's crying/fussing prior 24 h (≥3 cf. <3 h)	0.01	[−0.55 to 0.57]
Baby's sleep a problem in past 2 weeks (yes cf. no)	**2**.**44**	**[1.65–3.24]**
IBM Care score	−0.32	[−1.12 to 0.49]
IBM Control score	0.32	[−0.57 to 1.21]
Characteristics predicting change of PHQ-9 scores from baseline to follow-up (linear regression)		
Trial arm (intervention cf. control)	−0.27	[−0.63 to 0.08]
Age (in years)	−0.01	[−0.06 to 0.04]
Language spoken at home (English cf. other languages)	**0**.**89**	**[0.35–1.43]**
Education level (university degree cf. partial or complete secondary or certificate level)	−0.15	[−0.61 to 0.32]
Marital status (married cf. *de facto* or single)	−0.11	[−0.47 to 0.26]
Occupation (managerial or professional cf. other occupations)	−0.06	[−0.46 to 0.35]
Holds a health-care card (yes cf. no)	0.21	[−0.60 to 1.02]
VPS Vulnerability Subscale score	−0.02	[−0.09 to 0.05]
Past psychiatric illness (yes cf. no)	0.03	[−0.47 to 0.53]
Childhood physical/sexual abuse (yes cf. no)	−0.41	[−1.01 to 0.19]
Number of pregnancies (one cf. >1)	0.15	[−0.27 to 0.57]
Duration of baby's crying/fussing prior 24 h (≥3 cf. <3 h)	0.17	[−0.13 to 0.47]
Baby's sleep a problem in past 2 weeks (yes cf. no)	−**1**.**06**	[−**1.51 to −0.61]**
IBM Care score	−0.29	[−0.85 to 0.28]
IBM Control score	0.36	[−0.25 to 0.97]
**Fit indices**	**Estimates**	
RMSEA (probability RMSEA ⩽0.05)	0.04 (0.50)	
Comparative fit index	0.950	
Tucker–Lewis index	0.901	

a All regression coefficients were estimated simultaneously using Conditional Latent Growth Curve Model. Significant regression coefficients are in bold.

b Intercept.

c Slope.

In the GAD-7 growth model, women with higher VPS Vulnerability Subscale scores, and women whose babies had a sleep problem, had higher GAD-7 scores at baseline and greater reduction rates in these scores in the first 18 months after giving birth than other women. When baseline characteristics and these differences were controlled for, women in the intervention arm had significantly greater reduction rate in GAD-7 scores (generalised anxiety symptoms) than women in the control arm.

In the PHQ-9 growth model, women with higher VPS Vulnerability Subscale scores and women with a psychiatric history had higher baseline PHQ-9 scores (depression symptoms) than those without these characteristics, but there was no significant difference between these two groups in the rates of change in PHQ-9 scores from baseline to follow-up. Women whose baby had a sleep problem had higher baseline PHQ-9 scores and a significantly greater reduction rate in scores from baseline to follow-up than others. Women who spoke English as a first language had lower baseline PHQ-9 scores and less reduction from baseline to follow-up. Women in the intervention group had higher baseline PHQ-9 scores and a greater reduction rate in scores than women in the control arm, but this did not reach statistical significance.

In the sensitivity analyses, the transformation tests indicated that the square root was the appropriate transformation for both GAD-7 and PHQ-9 scores to become normally distributed variables. The growth models with the transformed outcomes confirmed all the significant associations found in the previous models (online Supplementary Tables S1 and S2).

The fit indices of the four Conditional Latent Growth Curve Models indicate that the models fit the data well.

### Magnitude of change in proportions scoring in the asymptomatic range

The magnitude in changes in women's mental health from baseline to follow-up was greater in the intervention than the control arm (Table 4). The proportion of women in the intervention arm having GAD-7 scores ⩽4 increased 12.9% (from 66.7% at baseline to 79.6% at follow-up) (*p* = 0.008) compared with a decrease of 2.0% in the control arm (from 76.3% to 74.3%, *p* = 0.690). The proportion of women in the intervention arm with PHQ-9 scores ⩽4 increased 5% (from 66.0% at baseline to 71.0% at follow-up, *p* = 0.339) in the intervention compared with 1.5% (from 71.1% to 72.4%, *p* = 0.799) in the control arm.

**Table 4.** PHQ-9 and GAD-7 scores of 314 women (152 in the control arm and 162 in the intervention arm) who provided complete data

	Baseline		Trial endline		Follow-up	
	Control	Intervention	Control	Intervention	Control	Intervention
PHQ-9 score, median [IQR]	2 [1–5]	3 [2–5]	3 [1–4.5]	2 [1–5]	3 [1–5]	3 [1–5]
PHQ-9 severity, *n* (%)						
None (score ⩽4)	108 (71.1)	107 (66.0)	114 (75)	116 (71.6)	110 (72.4)	115 (71.0)
Mild (5–9)	37 (24.3)	40 (24.7)	32 (21.1)	34 (21.0)	33 (21.7)	38 (23.4)
Moderate or severe (10+)	7 (4.6)	15 (9.3)	6 (3.9)	12 (7.4)	9 (5.9)	9 (5.6)
GAD score, median [IQR]	2 [1–4]	3 [1–6]	2 [1–5]	2 [1–5]	2 [0–5]	2 [1–4]
GAD severity, *n* (%)						
None (score ⩽4)	116 (76.3)	108 (66.7)	108 (71.1)	118 (72.9)	113 (74.3)	129 (79.6)
Mild (5–9)	30 (19.7)	41 (25.3)	37 (24.3)	30 (18.5)	33 (21.7)	24 (14.8)
Moderate or severe (10+)	6 (4.0)	13 (8.0)	7 (4.6)	14 (8.6)	6 (4.0)	9 (5.6)

Given the evidence in the PP analysis of trial outcomes (Fisher *et al*. 2016), we also examined whether there were differences in changes between women who received the partial (primary care from a WWWT-trained MCHN and print materials) compared with the full three-component intervention which included attending the seminar. There was an improvement of 4.8% (from 69.9% to 74.7%, *p* = 0.488) among women who received the partial and 5.1% (from 62% to 67.1%, *p* = 0.506) among women who received the full intervention in PHQ-9 scores ⩽4. The proportion of women with GAD-7 score ⩽4 improved 2.4% (from 77.1% to 79.5%, *p* = 0.706) among women receiving the partial intervention and 24.1% (from 55.7% to 79.8%, *p* = 0.043) among women who received the full intervention (Table 5).

**Table 5.** PHQ-9 and GAD-7 scores of 162 women in the intervention arm who provided complete data and received with the partial (83 women) or full (79 women) intervention

	Baseline		Trial endline		Follow-up	
	Partial	Full	Partial	Full	Partial	Full
PHQ-9 score, median [IQR]	3 [2–5]	4 [2–6]	2 [1–4]	2 [1–5]	3 [1–5]	3 [1–6]
PHQ-9 severity, *n* (%)						
None (score ⩽4)	58 (69.9)	49 (62.0)	63 (75.9)	53 (67.1)	62 (74.7)	53 (67.1)
Mild (5–9)	19 (22.9)	21 (26.7)	16 (19.3)	18 (22.8)	18 (21.7)	20 (25.3)
Moderate or severe (10+)	6 (7.2)	9 (11.3)	4 (4.8)	8 (10.1)	3 (3.6)	6 (7.6)
GAD score, median [IQR]	2 [0–4]	4 [2–6]	2 [1–4]	3 [1–6]	2 [0–4]	2 [1–4]
GAD severity, *n* (%)						
None (score ⩽4)	64 (77.1)	44 (55.7)	69 (83.1)	49 (62.0)	66 (79.5)	63 (79.8)
Mild (5–9)	15 (18.1)	26 (32.9)	10 (12.1)	20 (25.3)	11 (13.3)	13 (16.4)
Moderate or severe (10+)	4 (3.8)	9 (11.4)	4 (2.8)	10 (12.7)	6 (7.2)	4 (3.8)

## Discussion

This is the longest follow-up to date of participants in a randomised controlled trial of a universal intervention to prevent PCMD. There were high retention fractions at trial endline, and follow-up, of a systematically recruited community cohort of women who had given birth to a first baby 18 months earlier.

We acknowledge the limitations of this follow-up study that because of project resource constraints, and respect for participants’ time, there were restrictions on the data that could be collected. We ascertained symptoms of depression and anxiety among women (reported here), and detailed information about the early development of their young children (which are not reported here), but were not able to collect comprehensive data directly from or about interactions with the intimate partners. Mental health was assessed using standardised self-reported symptom instruments and not diagnostic interviews. We also acknowledge that in the trial, only about half the women who were eligible actually attended the seminar component of this new intervention, most because their partners were unwilling to participate. It remains uncommon in Australia for men to attend postnatal primary care and, our strategies of providing personalised invitations and making the seminar accessible by offering it on a Saturday, increased participation well beyond the usual level, but not to full participation.

Nevertheless, we believe that the data provide robust indications of the long-term impact of WWWT, a brief gender-informed psycho-educational programme provided within 8 weeks of giving birth.

### Anxiety symptoms

In the whole cohort at trial endline, when women were 6 months postpartum, the point prevalence of anxiety disorders (generalised anxiety, panic disorder with or without agoraphobia, social phobia, separation anxiety or adjustment disorder with anxiety) (22/359, 6.13%) was higher than of depressive disorders (major depressive disorder, adjustment disorder with depressed mood) with or without a co-morbid anxiety disorder (12/359, 3.34%). The most common experience was anxiety symptoms above the clinical cut-off scores, but not meeting diagnostic criteria (GAD-7 scores 5–9). Prevalence of these mild-to-moderate anxiety symptoms was significantly lower in the intervention (19.3%) than the control arm (24.3%; AOR 0.57, 95% CI 0.34–0.96). Receiving the full three-component WWWT programme was associated with reduced prevalence of anxiety disorders (3/89; 3.37%) compared with usual care (11/173; 6.35%) (Fisher *et al*. 2016). These follow-up data indicate that these differences continued, with a significantly steeper slope in reduction in GAD-7 scores among participants in the intervention than the control arm. Most striking was the very substantial increase in the proportion with no anxiety symptoms at long-term follow-up among women who received the full three-component intervention compared with those who received the partial intervention or the usual standard of care.

### Depressive symptoms

At trial endline, prevalence of depressive disorders in the intervention group (3/89; 3.37%) was similar to that in the control group (5/173; 2.89%) and there were no differences in sub-clinical depressive symptom scores (PHQ-9 scores 5–9). At the 18-month follow-up, there were non-significant differences in slope of reduction in depressive symptoms in favour of the intervention and a larger proportion of women in the intervention than the control arm had become asymptomatic. However, there were no differences in changes in proportions without symptoms from baseline to follow-up between the groups who received the full, compared with the partial intervention.

### Postulated mechanisms of action

The postulated mechanisms of action of the psychoeducational WWWT programme are to promote respectful relationships between intimate partners by reducing critical or controlling interactions, recognising and countering gender stereotypes about responsibility for domestic tasks, increasing empathic recognition and promoting problem-solving capabilities, including about sharing the unpaid workload fairly. Among the majority whose relationship quality was optimal [IBM Care Subscale score ⩾75th percentile and Control Subscale scores ⩽25th percentile of sample distribution (Wilhelm & Parker, 1988)] at baseline, fewer women in the intervention than the control arm reported emotionally abusive behaviours from their partners at trial endline (Fisher *et al*. 2016; Wynter *et al*. 2017).

The data provide further evidence that unsettled infant behaviours, in particular sleep problems, are a clear indicator of significantly heightened psychological needs among women who have recently given birth. They were associated with significantly higher baseline depressive and anxiety symptom scores. The postulated mechanisms of action in the WWWT programme are that teaching parents from the earliest weeks how to recognise their baby's tired cues, understand infant sleep needs, use effective settling strategies and reduce unsustainable sleep associations will have benefits for women's mood, fatigue and confidence. At trial endline, people whose babies had been unsettled at baseline were more likely than others to have adopted infant behaviour management strategies to promote more settled behaviours. The significantly greater decline in anxiety among them suggests that this skill might have been incorporated and applied long term.

Although, because of project constraints, precise mechanisms could not be assessed using observational measures, the data indicate that the proposed conceptualisation of mental health being influenced by day-to-day interactions among intimate family members is plausible and that these are potentially modifiable. We postulated (see Box 1) that mental health problems would be averted and confidence promoted by reducing the key risks identified by Brown and Harris (Brown & Harris, 1978) of experiences of humiliation and entrapment. These suggest that the opportunities provided in the seminar to learn how to recognise infant tired cues and to respond with contingent and effective soothing and settling strategies, and to understand each other's changed roles, responsibilities and needs and to seek shared solutions have been incorporated and led to alterations in day-to-day interactions.

These data provide further evidence that, as proposed by Boyce *et al*. (1991) more than two decades ago, personality characteristics in particular high interpersonal sensitivity or excessive need for approval, and limited capacity for assertiveness, increase vulnerability to postnatal mental health problems among women. The WWWT programme educates women about how to have increased agency in their day-to-day interactions with their partners and their babies and this might have contributed to the significant reduction in GAD-7 scores among women with high VPS Vulnerability scores at baseline.

Although women with a psychiatric history were more likely to have higher baseline depressive symptoms than women without this history, it is notable that this history was not associated with changes in either GAD-7 or PHQ-9 scores from baseline to follow-up. This suggests that the WWWT programme is useful to women regardless of psychiatric history and supports the value of offering a universal mental health promotion programme within a stepped model of care.

The predominant long-term impact of this gender-informed intervention is on anxiety, the most prevalent postpartum mental health problem rather than on depression. Anxiety and depression have symptoms in common and are not absolutely distinct psychological states. We speculate that the WWWT approach is particularly effective for anxiety because it promotes agency and active problem-solving.

It was useful but not sufficient for the small group of women who experienced depression. They might have had more complex personal predicaments. As we argued in reporting the trial outcomes (Fisher *et al*. 2016), universal approaches will not obviate the needs some people will have for more intensive individual interventions and treatment, or relationship counselling, but are an important component of stepped approaches to health promotion.

The lasting impact of the intervention indicates that the capacity of the primary care nursing workforce to respond to psychological needs can be enhanced with brief training and opportunity to implement a highly structured programme with a clear facilitator's guide and programme materials. At trial endline, MCHNs in the intervention arm indicated that their routine skills had been increased, in particular in including fathers in first-time parents’ groups, addressing interpersonal conflict between intimate partners directly, and recognising their own gender stereotypes and noting and countering these among their patients (Fisher *et al*. 2016). Many already taught structured management of unsettled infant behaviours, but 64% had increased attention to it in their provision of usual care. These data provide further evidence that the seminar facilitated by MCHNs is the most influential component of the intervention and that the WWWT ‘About Parents’ and ‘About Babies’ materials are an evidence-informed and effective resource for primary care (see Box 1).

There is only one long-term follow-up of a universal prevention intervention. MacArthur *et al*. (2003) followed trial participants up to 12 months postpartum and demonstrated that the benefits of the intensive home visiting programme for depressive symptoms were maintained. They provide further illustration of the benefits for women's mental health of receiving care from psychologically skilled primary care professionals. However, it is not feasible to implement intensive home visiting in routine care in the Australia health system, which provides one home visit, and up to nine further centre-based consultations for healthy-baby checks and immunisation over the first 2 years of the baby's life. The data suggest that women's reports of infant sleep problems should be responded to actively, including with guidance about evidence-informed infant behaviour management strategies and not just with the reassurance or normalisation that is common in the usual standard of care (Wynter *et al*. 2015).

Confirmation of these findings is now required in further community-based trials and there is a potential to investigate, in laboratory conditions, differences in the quality of interactions between intimate partners and between mothers and their infants who have and have not received the WWWT programme. Overall, these data provide further evidence that this gender-informed, cost-effective, psycho-educational programme, which can be integrated readily into routine primary care, does not require intensive home visiting and in which the most important component, the seminar, is offered to groups and not individuals, is clinically effective. It has facilitated lasting changes in day-to-day interactions among families in the intervention group who had participated in the in-person seminar. In population terms, to have increased the proportion of women without anxiety symptoms by nearly 25% with the benefit sustained throughout the first 18 postpartum months is a major public health benefit. This follow-up evidence indicates that implementation in routine care at scale is warranted. As the intervention is for couples, wider community education and social marketing are likely to be required to enable men to appreciate the essential worth of their participation in infant care and household work, and that the WWWT programme is likely to assist them to become confident and effective in these roles and responsibilities.

The WWWT programme has been translated and culturally adapted into Vietnamese, Sinhala, Japanese and Mandarin in close consultation with communities. There are inter-cultural differences in traditions about co-sleeping with infants, and the age of introducing complementary foods to the infant diet and gender-based roles and responsibilities. Nevertheless, in all these adaptations, the community groups emphasised that the ideas and skills in the programme were highly relevant to their needs. Overall, these findings suggest that this evidence-informed psycho-educational early parenting programme has potential global relevance as a perinatal mental health promotion strategy, which contributes to the call for evidence-informed programmes for primary care, and the Global Strategy for Women's, Children's and Adolescents’ Health (2016–2030).

## Supplementary material

For supplementary material accompanying this paper visit http://dx.doi.org/10.1017/gmh.2018.20. click here to view supplementary material
