# Supplementary material for: Gender-informed psycho-educational programme to promote respectful relationships and reduce postpartum common mental disorders among primiparous women: long-term follow-up of participants in a community-based cluster randomised controlled trial
Source: Glob Ment Health (Camb). 2018 Sep 25;5:e30. doi: 10.1017/gmh.2018.20 (PMC6236212; doi:10.1017/gmh.2018.20)
Supplement: Supplementary file 1 [file S2054425118000201sup001.docx]

Supplementary Table 1. Conditional Latent Growth Curve Model of women’s GAD-7 square root scores from 6 weeks to 18 months postpartum

|  | Coefficient^(c)^ | 95% CI |
| --- | --- | --- |
| **GAD-7 trajectories** |  |  |
| Mean GAD-7 square root score at 6 weeks postpartum ^(a)^ | **1.49** | **[0.74 ; 2.23]** |
| Change in GAD-7 square root score from 6 weeks to 18 months postpartum ^(b)^ | -0.07 | [-0.59 ; 0.45] |
| **Characteristics associated with GAD-7 square-root score at baseline (Linear regression)** | | |
| Trial arm (intervention cf. control) | **0.24** | **[0.08;0.4]** |
| Age (in years) | 0.01 | [-0.02;0.02] |
| Language spoken at home (only English cf. other languages) | -0.2 | [-0.47;0.08] |
| Education level (university degree cf. partial or complete secondary or certificate level) | 0.01 | [-0.17;0.19] |
| Marital status (married cf. de facto or single) | -0.03 | [-0.26;0.19] |
| Occupation (managerial or professional cf. other occupations) | -0.07 | [-0.23;0.1] |
| Holds a Health Care card (yes cf. no) | -0.04 | [-0.25;0.17] |
| VPS Vulnerability Subscale score | **0.11** | **[0.09;0.13]** |
| Past psychiatric illness (yes cf. no) | 0.2 | [-0.01;0.41] |
| Childhood physical/sexual abuse (yes cf. no) | 0.16 | [-0.16;0.48] |
| Number of pregnancies (one cf. > one) | -0.09 | [-0.27;0.1] |
| Duration baby’s crying/fussing prior 24 h (3 hours or more cf. less than 3 hours) | 0.09 | [-0.11;0.28] |
| Baby’s sleep a problem in past 2 weeks (Yes cf. no) | **0.66** | **[0.44;0.88]** |
| IBM Care score | -0.01 | [-0.21;0.19] |
| IBM Control score | 0.2 | [-0.01;0.41] |
| **Characteristics predicting change of GAD-7 square-root scores from baseline to follow up (Linear regression)** | | |
| Trial arm (intervention cf. control) | **-0.14** | **[-0.26;-0.02]** |
| Age (in years) | 0.01 | [-0.01;0.02] |
| Language at home (English cf. others) | 0.05 | [-0.08;0.19] |
| Education level (university degree cf. partial or complete secondary or certificate level) | -0.02 | [-0.16;0.13] |
| Marital status (married cf. defacto or single) | 0.03 | [-0.11;0.17] |
| Occupation (managerial or professional cf. other occupations) | 0.06 | [-0.06;0.18] |
| Holds a Health Care card (yes cf. no) | 0.16 | [-0.02;0.35] |
| VPS Vulnerability Subscale score | **-0.03** | **[-0.04;-0.02]** |
| Past psychiatric illness (yes cf. no) | 0.11 | [-0.03;0.25] |
| Childhood physical or sexual abuse (yes cf. no) | -0.06 | [-0.29;0.17] |
| Number of pregnancies (one cf. > one) | 0.05 | [-0.09;0.18] |
| Duration baby’s crying/fussing prior 24 h (3 hours or more cf. less than 3 hours) | 0.01 | [-0.1;0.12] |
| Baby’s sleep a problem in past 2 weeks (yes cf. no) | **-0.23** | **[-0.35;-0.1]** |
| IBM Care score | -0.01 | [-0.13;0.12] |
| IBM Control score | -0.08 | [-0.27;0.12] |
| **Fit indices** | **Estimates** |  |
| RMSEA (Probability RMSEA <= .05) | <0.001 (0.99) |  |
| Comparative Fit Index | 1.00 |  |
| Tucker-Lewis Index | 1.02 |  |

^(a)^Intercept; ^(b)^ Slope; ^(c)^All regression coefficients were estimated simultaneously using Conditional Latent Growth Curve Model. Significant regression coefficients are in bold.

Supplementary Table 2. Conditional Latent Growth Curve Model of women’s PHQ-9 square root scores from 6 weeks to 18 months postpartum

|  | Coefficient^(c)^ | 95% CI |
| --- | --- | --- |
| **PHQ-9 trajectories** |  |  |
| Mean PHQ-9 square root score at 6 weeks postpartum ^(a)^ | **1.69** | **[1.12 ; 2.26]** |
| Change in PHQ-9 square root score from 6 weeks to 18 months postpartum ^(b)^ | -0.03 | [-0.40 ; 0.31] |
| **Characteristics associated with PHQ-9 square root scores at baseline (Linear regression)** | | |
| Trial arm (intervention cf. control) | 0.14 | [-0.01;0.29] |
| Age (in years) | 0.01 | [-0.02;0.02] |
| Language spoken at home (only English cf. other languages) | **-0.29** | **[-0.48;-0.11]** |
| Education level (university degree cf. partial or complete secondary or certificate level) | -0.01 | [-0.15;0.14] |
| Marital status (married cf. defacto or single) | 0.09 | [-0.11;0.3] |
| Occupation (managerial or professional cf. other occupations) | -0.03 | [-0.17;0.12] |
| Holds a Health Care card (yes cf. no) | 0.17 | [-0.09;0.42] |
| VPS Vulnerability Subscale score | **0.06** | **[0.04;0.08]** |
| Past psychiatric illness (yes cf. no) | **0.2** | **[0.03;0.38]** |
| Childhood physical/sexual abuse (yes cf. no) | 0.23 | [0.01;0.45] |
| Number of pregnancies (one cf. > one) | -0.1 | [-0.24;0.04] |
| Duration baby’s crying/fussing prior 24 h (3 hours or more cf. less than 3 hours) | 0.07 | [-0.06;0.2] |
| Baby’s sleep a problem in past 2 weeks (Yes cf. no) | **0.68** | **[0.5;0.86]** |
| IBM Care score | -0.13 | [-0.31;0.06] |
| IBM Control score | 0.04 | [-0.17;0.26] |
| **Characteristics predicting change of PHQ-9 square root** **scores from baseline to follow up (Linear regression)** | | |
| Trial arm (Intervention cf. control) | -0.06 | [-0.16;0.04] |
| Age (in years) | -0.01 | [-0.02;0.01] |
| Language spoken at home (English cf. other languages) | **0.15** | **[0.02;0.28]** |
| Education level (university degree cf. partial or complete secondary or certificate level) | -0.04 | [-0.16;0.08] |
| Marital status (married cf. defacto or single) | -0.02 | [-0.13;0.1] |
| Occupation (managerial or professional cf. other occupations) | 0.01 | [-0.11;0.12] |
| Holds a Health Care card (yes cf. no) | -0.01 | [-0.24;0.23] |
| VPS Vulnerability Subscale score | -0.01 | [-0.02;0.01] |
| Past psychiatric illness (yes cf. no) | 0.03 | [-0.1;0.17] |
| Childhood physical/sexual abuse (yes cf. no) | -0.1 | [-0.29;0.09] |
| Number of pregnancies (one cf. > one) | 0.06 | [-0.06;0.18] |
| Duration baby’s crying/fussing prior 24 h (3 hours or more cf. less than 3 hours) | 0.02 | [-0.06;0.1] |
| Baby’s sleep a problem in past 2 weeks (yes cf. no) | **-0.29** | **[-0.4;-0.19]** |
| IBM Care score | -0.04 | [-0.19;0.11] |
| IBM Control score | 0.1 | [-0.06;0.25] |
| **Fit indices** | **Estimates** |  |
| RMSEA (Probability RMSEA <= .05) | 0.01 (0.89) |  |
| Comparative Fit Index | 0.98 |  |
| Tucker-Lewis Index | 0.96 |  |

1. Intercept; ^(b)^ Slope; ^(c)^All regression coefficients were estimated simultaneously using Conditional Latent Growth Curve Model. Significant regression coefficients are in bold.
